# Burnout and training satisfaction of medical residents in Greece: will the European Work Time Directive make a difference?

**DOI:** 10.1186/1478-4491-8-16

**Published:** 2010-07-01

**Authors:** Pavlos Msaouel, Nikolaos C Keramaris, Athanasios Tasoulis, Dimitrios Kolokythas, Nikolaos Syrmos, Nikolaos Pararas, Eleftherios Thireos, Christos Lionis

**Affiliations:** 1 Greek Junior Doctors and Health Scientists Society, Greece; 2 Athens Medical Society, Athens, Greece; 3 Clinic of Social and Family Medicine, School of Medicine, University of Crete, Greece

## Abstract

**Background:**

The aim of this study is to determine the prevalence of burnout in Greek medical residents, investigate its relationship with training satisfaction during residency and survey Greek medical residents' opinion towards the European Work Time Directive (EWTD).

**Methods:**

A Multi-centre, cross-sectional survey of Greek residents was performed. The Maslach Burnout Inventory (MBI) was used to measure burnout, which was defined as high emotional exhaustion, combined with high depersonalization or low personal accomplishment. In addition, seven questions were designed for this study to evaluate self-reported resident training satisfaction and three questions queried residents' opinion on the EWTD and its effects on their personal and social life as well as their medical training. Univariate, bivariate and multivariate statistical models were used for the evaluation of data.

**Results:**

Out of 311 respondents (77.8% response rate), 154 (49.5%) met burnout criteria and 99 (31.8%) indicated burnout on all three subscale scores. The number of residents that were dissatisfied with the overall quality of their residency training were 113 individuals (36.3%). Only 32 residents (10.3%) believed that the EWTD implementation will not have any beneficial effects for them.

**Conclusions:**

Both burnout and training dissatisfaction were common among Greek residents. Systemic interventions are thus required within the Greek health system, aimed at reducing resident impairment due to burnout and at improving their educational and professional perspectives. Although residents' opinion on the EWTD was not associated with burnout levels, the EWTD was found to be predominantly supported and anticipated by Greek residents and should be implemented to alleviate their workload and stress.

## Background

### Resident burnout

Residency is a stressful stage of physician training during which residents work for long hours while their responsibility towards the lives of patients simultaneously increases, ideally along with their knowledge and experience. However, this is not always the case: accumulation of responsibilities disproportional to residents' knowledge and experience can be rather common and, combined with physical exhaustion, can set the scene for burnout [1-3]. Resident burnout has been associated with worse health status, substance abuse, tendency towards depression, higher anxiety and suicidal thoughts [1,2,4-7]. Nevertheless, two extensive reviews of the literature by Thomas (2004) [1] and by Prins et al. (2007) [2] demonstrated the surprising scarcity of published data on resident burnout. Particularly among European states, very few reports on resident burnout have thus far been published [5,8-13]. The syndrome's repercussions do not solely affect the giving end of the health care process (health workers) but the receiving end as well; particularly, the users of health services: patients and society as a whole. Therefore, high levels of burnout in doctors can lead to poor performance, medical errors, decreased quality of medical care and deterioration of confidence in health services [1,2,4,12,14-17].

The Greek situation may be even more complex as a consequence of the peculiarities of the Greek National Health System (NHS) [18-21]. More than twenty-five years after its foundation in 1983, the Greek NHS faces a manifold crisis (financial and organizational) and is in need of urgent reforms [20,22-26]. The medical education system is outdated [27-31], the European Work Time Directive (EWTD) is still not implemented and Greek junior doctors face the risk of unemployment due to the overwhelming inflation of medical graduates [32]. We have previously shown that such discouraging conditions prompt an alarming percentage (70.3%) of Greek medical students to consider entering a medical residency abroad [33].

The organizational difficulties particular to the Greek NHS [18-26] may result in increased work stress, role ambiguity and higher uncertainty about the future prospects of residents. The underdevelopment of Greek integrated primary health care [22] intensifies the workload of residents working in secondary institutions. Furthermore, the widespread use of informal payments within the Greek NHS [23], the growing imbalance between the Greek public and private health sector [25] as well as the perceived inefficiency and inequity of Greek health care [18,20,24,25] result in increased patient dissatisfaction which may aggravate conflicts between patients and residents. Higher work-related stress, work role problems, anxiety about the future, work overload and confrontations with patients have previously been associated with burnout [1,2,34-36] and may thus render those residents who do choose to train in Greece vulnerable to this syndrome.

### The EWTD in Greece and its potential impact on residency training

The EWTD regulates the working time schedule, the annual paid leave, the characteristics of night shift duties and the time to rest after on-call duty. European Member States were thus obliged to gradually reduce the working hours of residents to no more than 48 hours per week by 1 August 2009 [37]. This directive was incorporated in Greek Legislation by the presidential edict 76/05 (2005). However, at the time of writing and the latest revision of this manuscript (June 2010), the new working hour limits have yet to be implemented in Greece.

It should be noted that concerns have been expressed regarding the EWTD effects on resident training, especially on surgical residents who may experience reductions in the number of didactic outpatient and operative sessions, as well as discontinuity of presence during clinical and postoperative care [38-42]. Such problems may be more pronounced in Greece due to the notable limitations of structured resident training [27-30]. Particularly in the surgical residencies, log-books have not been formally adopted by all training centers, the residency curricula among the various clinics have not been systematized and significant heterogeneity in quality of the various training programs exists [29,31]. The Greek educational system is thus largely based on the informal practical training of younger residents by those most experienced. Decreasing the active working hours may reduce clinical and operative experience as well as significantly limit the time available for senior residents or specialists to teach younger residents [29].

### Objectives

The overall aim of this study is to report the prevalence of burnout syndrome among Greek medical residents. In addition, Greek residents' satisfaction and opinion on aspects of their training are investigated. This study also reports on Greek residents' attitudes towards the EWTD as well as the demographic and work-related factors that are associated with these opinions. Furthermore, the present study aims to delineate the relationship of burnout scores with Greek residents' demographic characteristics, residency field, future outlook, self-reported training satisfaction and subjective perceptions towards the EWTD.

## Methods

### Participants and data collection

Eight major Greek hospitals, all with a capacity of over 200 beds, representing three large Greek cities (Athens/Piraeus, Heraklion and Volos) and covering 30 different specialties were selected for this cross-sectional study. The Greek system does not allow official residency training in private hospitals and therefore all institutions surveyed in this study were public. Every resident was given a number according to the official enrolment lists that had been kindly provided by each of the hospitals. Out of 1407 eligible residents in all hospitals, 400 (50 from each hospital) were randomly selected by a computerized method to participate in the sample, a number that represents approximately 4% of the total number of residents training in Greece [43]. Attendance was elective and all participants were informed that responses would be anonymous and were blinded to the scope and purpose of the study. The residents were asked to return the completed questionnaires to a sealed box provided in each hospital.

### Sample size

Our survey used a two-stage cluster sampling design and a design effect value of 2.0 was thus adopted. There are currently approximately 10 300 residents training in Greece [43]. A minimum sample size of 296 was thus required for this survey to represent the population of Greek residents at the 95% confidence level and a confidence interval of 8. The questionnaire was administered to 400 residents and 311 completed questionnaires were returned and included in this study.

### Survey measures

The first 9 questions queried the socio-demographic profile, specialty choices and the respondents' opinion on their future employment. We combined residencies according to their conceptual and occupational relations and formed four different medical 'fields': a. internal medicine (n = 108; paediatrics and neurology were also included in this group) b. surgical specialties (n = 135) c. diagnostic and laboratory specialties (n = 45) and d. general practice (n = 23). The second part of the questionnaire was devoted to medical training quality. These questions were developed based on a list of factors related to residents' satisfaction with the quality of their training programs that were identified by a review of the relevant literature [44-46]. Residents were asked to rate their opinion of seven statements regarding their residency training on a 5-point Likert scale ranging from "strongly disagree" (score of 1) to "strongly agree" (score of 5). The third part consisted of three multiple choice questions ("Yes", "No" or "I do not know/do not have an opinion") querying residents' opinion on the EWTD and its effects on their personal and social life and their medical training.

The last part of the survey assessed resident burnout using the Maslach Burnout Inventory (MBI) [47] which is the most established and widely used measure of burnout among physicians [1]. The MBI is a 22-item questionnaire that evaluates the 3 domains of burnout: emotional exhaustion (measured by 9 items), depersonalization (measured by 5 items) and lack of personal accomplishment (measured by 8 items) [47]. Respondents rate the frequency of burnout symptoms on a 7-point Likert-scale ranging from 0 (never) to 6 (daily) and independent scores are calculated for each of the 3 components of burnout. Each component score was categorized as "low", "moderate" or "high" based on previous results on 1104 medical professionals [47]. Thus, scores on emotional exhaustion of ≥ 27 were considered high, of 19-26 moderate, and of ≤ 18 low. Depersonalization scores of ≥ 10 were considered high, of 6-9 moderate and of ≤ 5 low. Personal accomplishment scores of ≤ 33 were considered low, of 34-39 moderate and of ≥ 40 high. A high classification on the emotional exhaustion or depersonalization subscales relates to higher professional burnout, while the personal accomplishment subscale has an inverse relationship to burnout with lower personal accomplishment scores indicating higher professional burnout [47]. According to previously established criteria [48] considered to be the most accurate in obtaining a dichotomous burnout score [2,48], resident responses with a high score on the emotional exhaustion subscale combined with a high score on depersonalization or a low score on personal accomplishment were classified as having burnout. The Greek translation of the MBI has been previously validated [49] and used among Greek health care professionals [50-52].

### Statistical analysis

Variables were maintained as continuous or categorical according to their original form in the questionnaire. The Kolmogorov-Smirnov test was applied for analysis of variance in all continuous variables. The choice of methods for statistical testing of continuous variables was based on whether the data permitted parametric or non-parametric analysis. Categorical variables were compared using Pearson's chi-square tests. Multivariate analysis was performed using linear regression analysis. Reliability of the three MBI subscales was determined using Cronbach's alpha. A minimum alpha value of 0.70 is generally desirable [53]. A *P*-value of < 0.05 was considered statistically significant. *P-*values of post hoc paired comparisons, following Kruskal Wallis or chi-square tests, were adjusted with the Bonferroni method.

## Results

### Demographic data

Table 1 lists the respondents' demographic profile and specialty. A total of 311 completed questionnaires were returned (response rate 77.8%, 172 males, 139 females, median age 32 years). Respondents' demographic characteristics were similar across the 8 hospitals and the four medical fields surveyed (Kruskal-Wallis one-way analyses of variance and Pearson's chi-square tests, all *P*-values were > 0.1).

**Table 1:** Resident demographic profile and specialty.

1. Age	Median: 32 years		2. Sex^a^	Male	Female	
	Range: 26-45 years			172 (55.3%)	139 (44.7%)	
**3. Hospital^b^**	Achillopouleio General Hospital of Volos	Agios Savvas Hospital	General Hospital of Athens "Ippokrateio"	General Hospital of Athens "Laiko"	Metaxa Hospital	Nikaia General Hospital
	38 (56.7%)	42 (31.3%)	45 (22.1%)	10 (3.9%)	44 (31.2%)	33 (13.6%)
	Tzaneio Hospital	Venizeleio General Hospital				
	51 (25%)	48 (30.2%)				

**4. Marital Status^a^**	Married or partnered	Single	**5. Have children**^**a**^	Yes	No	
	107 (34.4%)	204 (65.6%)		50 (16.1%)	10 (83.9%)	

**6. Resident**	Anaesthesiology	Cardiology	Cytology	Endocrinology	Gastroenterology	General Practice
**speciality **^**c**^	13 (2.8%)	14 (2.5%)	4 (4.9%)	4 (4.6%)	8 (6.5%)	23 (3.3%)
	General Surgery	Haematology	Internal Medicine	Medical Microbiology	Nephrology	Neurology
	50 (4.4%)	4 (3.2%)	39 (2.7%)	12 (1.7%)	4 (2.6%)	6 (2.6%)
	Neurosurgery	Nuclear Medicine	Obstetrics and gynaecology	Oncology	Ophthalmology	Orthopaedics
	8 (8.6%)	4 (6%)	10 (2.3%)	7 (13.2%)	8 (2.6%)	14 (2.5%)
	Otolaryngology	Pathology	Paediatric Surgery	Paediatrics	Plastic Surgery	Psychiatry
	11 (4.8%)	5 (3.1%)	3 (5.0%)	14 (2.5%)	5 (8.6%)	5 (1.6%)
	Pulmonary Medicine	Radiation Oncology	Radiology	Thoracic Surgery	Urology	Vascular Surgery
	7 (2.3%)	5 (9.4%)	11 (1.9%)	3 (3.9%)	5 (2.4%)	5 (10.6%)

^a ^Percentages in parentheses represent the rate per cent to the study's 311 respondents.

^b ^Percentages in parentheses represent the rate per cent to each hospital's total residents.

^c ^Percentages in parentheses represent the rate per cent to the total number of residents currently in training in each specialty in Greece [43].

### Resident burnout

Reliability analysis of each MBI subscale showed acceptable alpha values: 0.85 for emotional exhaustion; 0.81 for depersonalization and 0.78 for personal accomplishment. Table 2 shows the distribution of residents in each MBI subscale category. Notably, 154 residents (49.5%) met the criteria for overall burnout and 99 of the respondents (31.8%) satisfied all three subscale scores for high burnout (high emotional exhaustion, high depersonalization and low personal accomplishment).

**Table 2:** Resident Burnout, Future Outlook, Opinion on Residency Training and on the European Work Time Directive.

Future outlook^a^					
Do you believe it will be hard finding/keeping a job as a physician in the future?	Yes	No	I don't Know		
	145 (46.6%)	73 (23.5%)	93 (29.9%)		
**Opinion on medical residency training^a, b^**	Disagree (scores of 1 or 2)	Neither agree nor disagree (score of 3)	Agree (scores of 4 or 5)	Average Score	Range
My supervisors care about my residency training	131 (42.1%)	87 (28%)	93 (30%)	2.75	1-5
I am satisfied with the overall quality of CME I have participated in during my residency training	130 (41.8%)	110 (35.4%)	71 (22.8%)	2.67	1-5
I do too much scutwork/paperwork during my residency	63 (20.3%)	56 (18%)	192 (61.7%)	3.69	1-5
I dedicate considerable time on patient care	93 (29.9%)	79 (25.4%)	139 (44.7%)	3.16	1-5
I am satisfied with the responsibilities and time given to me for patient care	115 (37%)	110 (35.4%)	86 (27.7%)	2.82	1-5
I am satisfied with the level of teamwork and support from my peers	55 (17.7%)	79 (25.4%)	177 (56.9%)	3.5	1-5
I am satisfied with the overall quality of training in my clinic	113 (36.3%)	124 (39.9%)	74 (23.8%)	2.76	1-5

**Opinion on the European Work Time Directive (EWTD)^a^**	Yes	No	I don't Know		
Implementing the EWTD is necessary	233 (74.9%)	39 (12.5%)	39 (12.5%)		
The EWTD will be beneficial to my personal and social life	231 (74.3%)	44 (14.1%)	36 (11.6%)		
The EWTD will have positive effects on the quality of my residency training	174 (55.9%)	55 (17.7%)	82 (26.4%)		

	Categorization				
			
**Maslach Burnout Inventory Scores^c^**	High	Moderate	Low	Average Score	Range
Emotional Exhaustion	176 (56.6%)	76 (24.4%)	59 (19%)	27.9	0-54
Depersonalization	206 (66.2%)	39 (12.5%)	66 (21.2%)	12.2	0-30
Personal Accomplishment	55 (17.7%)	74 (23.8%)	182 (58.5%)	31.6	7-48

^a ^Percentages in parentheses represent the rate per cent to the study's 311 respondents.

^b ^Responses were rated on 5-point Likert scale ranging from "strongly disagree" (score of 1) to "strongly agree" (score of 5).

^c ^Respondents rated the frequency of burnout symptoms on a 7-point Likert-scale ranging from 0 (never) to 6 (daily) and independent scores are calculated for each of the 3 components of burnout. Percentages in parentheses represent the rate per cent to the study's 311 respondents. A high classification on the emotional exhaustion or depersonalization subscales relates to higher professional burnout, while lower personal accomplishment scores indicate higher professional burnout

### Future outlook

A considerable portion of respondents (46.6%) were unoptimistic about their future employment prospects as physicians and 29.9% were oblivious to what their future might bring (Table 2). These proportions were not significantly affected by the respondents' medical field or demographic variables (Kruskal-Wallis one-way analyses of variance and Pearson's chi-square tests, *P*-values > 0.1) with the exception of hospital of residency. More specifically, 32 respondents training at one General Hospital of Crete (66.7%) believed that it will be hard to find or keep a job as a physician in the future, a percentage that is significantly higher compared to responses from all other hospitals (Pearson's chi-square tests, *P *< 0.05 following adjustment with the Bonferroni method).

### Training satisfaction

As shown in Table 2, a substantial proportion of residents believed that their supervisors are indifferent towards their training and were dissatisfied with the overall quality of training in their clinic and with their continuous medical education (CME) activities. Furthermore, approximately 3 in every 5 Greek residents asserted that their daily residency schedule is hampered by too much 'scutwork' (menial tasks). On the other hand, a sizable proportion of residents (44.7%) did feel that they dedicate a considerable amount of time to their patients' care, yet a notable fraction of these residents (22.3%) were dissatisfied with the patient care responsibilities that they are administered, and only 43.1% felt that the time they invested in patient care was well spent. On a more positive note, more than half of the residents (56.9%) conveyed their satisfaction with the level of support and cooperation with their peers (Table 2). Residents' gender, marital status and parenthood did not significantly modify any of the satisfaction scores (Kruskal-Wallis one-way analyses of variance, *P*-values > 0.05) while age significantly correlated (Spearman rank correlation), albeit weakly, with only two variables, i.e. older residents were more likely to be dissatisfied with peer interactions (r = -0.121, *P *= 0.033) and less likely to feel that they are doing too much 'scutwork' (r = -0.142, *P *= 0.012).

Residents' opinions on their supervisors' interest towards their training, on the merit of their CME activities, on the responsibilities and time given to them for patient care and on the overall quality of training in their clinic were similar between the four medical fields (Kruskal-Wallis one-way analyses of variance, *P*-values > 0.1). However, surgical residents were less likely to report satisfaction with peer interactions compared to the other groups (Mann-Whitney U tests, *P*-values were < 0.01 following adjustment with the Bonferroni method). Furthermore, those belonging in the diagnostic and laboratory specialties group were less likely to report excessive 'scutwork' during their residency training compared to those training in the internal medicine, surgical or general practice fields (Mann-Whitney U tests; *P*-values were < 0.05 following adjustment with the Bonferroni method).

### Opinion on the EWTD

The predominant opinion among medical residents training in Greece is that implementing the EWTD is a necessary step (74.9%) and that the EWTD will be beneficial to their personal and social life (74.3%). In comparison, the percentage of residents who feel that the EWTD will have positive effects on their quality of training was lower (55.9%) but still represented more than half of the total responses (Table 2). Only 32 residents (10.3%) stated that the EWTD will not have beneficial effects on any aspect of their life. Pearson's chi-square tests did not reveal any significant differences (*P*-values > 0.1) on EWTD attitudes between the four medical fields. Likewise, residents' age, gender, marital status, parenthood and hospital of training did not significantly modify any of the EWTD responses (Pearson's chi-square tests and Kruskal-Wallis one-way analyses of variance, all *P*-values > 0.05).

### Factors related to resident burnout

Respondents' demographics, residency field and opinions on the EWTD did not correlate significantly with any of the MBI subscales (Spearman rank correlations, Kruskal-Wallis one-way analyses of variance and Mann-Whitney U tests, all *P*-values > 0.05). Due to Greek hospitals varying in specialties for residency training they offer, these two variables are not independent of each other and may thus confound interpretations of their potential relationship to burnout. Multivariate linear regression analyses were performed to adjust for this potential confounding effect. The linear regression models showed that neither residents' hospital of training nor their residency field affected the MBI subscale scores (*P*-values > 0.05). Residents' opinion on their future prospects did not affect their depersonalization and personal accomplishments scores (Kruskal-Wallis one-way analyses of variance, all *P*-values > 0.1). However, residents who believed that it will be difficult to find employment as physicians in the future demonstrated significantly higher emotional exhaustion (Mann-Whitney U tests, *P*-values were < 0.05 following adjustment with the Bonferroni method), compared to those who were unsure or those who did not have such concerns.

The correlation coefficients (Spearman rank correlations) between each MBI subscale and residents' opinions on aspects of their residency training are presented in Table 3. The strongest correlations noted were those between personal accomplishment and satisfaction with colleague support/teamwork (r = +0.323, *P *< 0.001) as well as between personal accomplishment and time spent on patient care (r = +0.275, *P *< 0.001). Interestingly, more time in patient-care activities also resulted in increased emotional exhaustion of residents (r = +0.116, *P *= 0.041). Despite being statistically significant, the correlation coefficients were weak indicating that the variable pairs do not fully represent the same construct.

**Table 3:** Correlation between the Maslach Burnout Inventory subscales and residents' opinions on their residency training (n = 311).

	**Emotional exhaustion**^**b**^	**Depersonalization**^**b**^	**Personal accomplishment**^**b**^
**My supervisors care about my residency training**^**a**^	r = -0.124	r = -0.133	r = +0.224
	P = 0.029^c^	P = 0.019^c^	P < 0.001^c^
**I am satisfied with the overall quality of CME I have participated in during my residency training**^**a**^	r = -0.060	r = -0.150	r = +0.177
	P = 0.289	P = 0.008^c^	P = 0.002^c^
**I do too much scutwork/paperwork during my residency^a^**	r = +0.218	r = +0.088	r = -0.252
	P < 0.001^c^	P = 0.122	P < 0.001^c^
**I spend considerable time on patient care^a^**	r = +0.116	r = -0.031	r = +0.275
	P = 0.041^c^	P = 0.59	P < 0.001^c^
**I am satisfied with the responsibilities and time given to me for patient care**^**a**^	r = -0.087	r = +0.012	r = +0.173
	P = 0.127	P = 0.831	P = 0.002^c^
**I am satisfied with the level of teamwork and support from my peers**^**a**^	r = -0.113	r = -0.214	r = +0.323
	P = 0.046^c^	P < 0.001^c^	P < 0.001^c^
**I am satisfied with the overall quality of training in my clinic**^**a**^	r = -0.089	r = -0.071	r = +0.147
	P = 0.118	P = 0.213	P = 0.009^c^

^a ^Responses were rated on a 5-point Likert scale ranging from "completely disagree" (score of 1) to "completely agree" (score of 5).

^b ^Respondents rated the frequency of burnout symptoms on a 7-point Likert-scale ranging from 0 (never) to 6 (daily) and independent scores are calculated for each of the 3 components of burnout. Higher scores on the emotional exhaustion or depersonalization subscales relates to higher professional burnout, while lower personal accomplishment scores indicate higher professional burnout

^c ^Statistically significant correlation (P < 0.05)

## Discussion

The present, multi-institutional study is to our knowledge the largest published study of burnout among Greek physicians in general and medical residents in particular. Burnout was found to be highly prevalent in Greek residents with approximately one half of respondents meeting the criteria for burnout and 3 in 10 medical residents scoring high in all domains of burnout. These results are more than two-fold higher compared to data from other European Union countries [5,8,54]. A number of factors specific to the Greek health care system may help explain this discrepancy. Health care in Greece is provided by the country's NHS, by the private sector and by certain public insurance organizations, including the National Foundation for Social Insurance (IKA), which function as bilateral monopolies similarly to the U.S.A. Health Maintenance Organizations (HMOs). A strong lack of coordination between these alternative forms of health care delivery has generated a number of challenging structural problems in the Greek health sector [18]. Furthermore, general practice and primary and preventive care are severely underdeveloped in Greece [22]. Notably, a recent study has demonstrated the need for a 619% increase in Greek generalist physicians in order to alleviate the current deficiencies in primary health care [55]. Consequently, the on-call personnel of secondary institutions are encumbered by exhaustive workloads that can aggravate resident burnout levels.

Various studies have examined the relationship between resident burnout and demographic factors, showing weak or negative associations [1,2]. These data, although often limited by small sample sizes, indicate that individual demographic factors may not be consistently associated with resident burnout [1]. Indeed, the present study did not find any significant relationships between burnout subscales and respondents' gender, marital status, age and parenthood. It should be noted that higher depersonalization scores among male respondents, when compared to female respondents, has been a very common finding among previous studies [2,8,35,56]. However, other studies, particularly those conducted in a Greek setting, have reported no significant association between depersonalization and gender among health professionals [52,57-59]. Similar results have been reported in other Greek populations, including a study of 200 elementary teachers [60] as well as a validation study of the MBI-Educators Survey in 771 Greek Cypriot teachers [61]. The results of the present study, based on the largest sample of Greek physicians reported to date, are consistent with the above data and may indicate the influence of cross-cultural variation or of other unknown factors on the relationship between gender and burnout scores.

In agreement with a previous study, specifically reporting on the effect of resident specialty on burnout scores [16], residency field did not affect burnout scores among Greek residents. However, further research using larger sample sizes will be required to corroborate this finding. Furthermore, no difference between resident burnout levels was found among the Greek hospitals surveyed (all with > 200-bed capacity), indicating that residency in large Greek hospitals will equally predispose a young physician to burnout.

It is of note that almost half of the present study's respondents believed that their future professional perspectives as medical doctors were dim, irrespective of their medical specialty or other demographic variables, with the exception of hospital of training. More specifically, residents training at one general hospital located in the city of Heraklion (Crete) were significantly more likely to be unoptimistic about their future employment prospects compared to their peers from any of the other hospitals surveyed. The present study did not ask respondents about a number of individual, familial, cultural, economic and workplace determinants which may influence self-perceived job insecurity [62,63]. Such unmeasured potential confounders may explain the observed institutional difference in this study and should be explored in future research. Self-reported job insecurity has been associated with lower mental and physical health [64,65]. Previous studies have reported higher rates of burnout among internal medicine residents feeling uncertain about their future [4] as well as orthopaedic residents who had increased anticipation of debt at the end of training [66]. Accordingly, it was presently shown that Greek residents feeling uncertain about their future working prospects were significantly more likely to be emotionally exhausted, although it should be noted that no effects on depersonalization or personal accomplishment were observed.

A considerable proportion of Greek residents were found to be dissatisfied with the quality of their residency training, CME activities, work responsibilities and supervisors' interest towards their training. Furthermore, the majority of residents felt that their daily schedule involved too much routine and trivial medical paperwork. It should be noted that these subjective perceptions were significantly associated, albeit weakly, with burnout subscales and may thus provide potential targets for systemic interventions. In addition, a large number of Greek medical residents were satisfied with peer interaction and support and this parameter also positively correlated with better personal accomplishment scores. Therefore, such reciprocal relationships should both be maintained and further encouraged.

A striking finding of this study was the predominant demand for the EWTD by Greek residents, regardless of specialty. Not even the valid concerns posed by surgical residents in other European Union countries [38-42] could attenuate this strong appeal for limiting the working hours of Greek junior doctors. However, no association was noted between Greek residents' opinion towards the EWTD and burnout. Previous research has not shown a significant relationship between resident work hours and burnout scores [9,67]. Moreover, Gelfand et al.[68] found that decreasing resident working hours from 100.7 to 82.6 per week did not significantly affect burnout rates of surgical residents. Taken together, these data suggest that the ETWD implementation will likely improve personal and social aspects of Greek residents' life but may not affect burnout levels per se. Thus, further interventions will be necessary to prevent burnout among residents in Greece.

A comparative analysis between the present study's burnout scores in Greek residents and previous data on Greek doctors [50-52] is shown in Table 4. Significantly higher burnout rates were observed in Greek residents surveyed in the present study compared to Greek internal medicine specialists and Greek healthcare employees. It is generally expected that fully trained specialists should feel more accomplished compared to physicians still in training. However, Greek general practice residents showed significantly higher personal accomplishment scores compared to the data on Greek general practitioners reported by Soler et al. (Table 4) [50]. General practice has a number of unique characteristics compared to other medical specialties in Greece, which may explain the declining over time of subjective personal accomplishment levels. The role of general practice within the Greek NHS is underecognised, and general practitioners are not highly regarded by other specialists [69,70]. Furthermore, the general practice residency curriculum is fundamentally different compared to all other medical specialties in Greece [71]. It consists of 4 years of training with only the final 10 months being spent in a primary health care center where residents work with and are trained by general practice specialists [71]. During the first 3 years, general practice residents rotate through various residency specialty programs, beginning with 6 months of internal medicine, where they are grouped and trained as indistinguishable members of each program's medical team. It is during the final 10 months that they are practically introduced to the challenges and esteem issues peculiar to general practice, and at this point they may begin to further question their own competence and performance [69,70,72,73]. The present study was not conducted in a primary health center and it is therefore conceivable that the general practice residents surveyed have not yet been exposed first-hand to the low esteem held by other specialists towards the work carried out by general practitioners. It should also be noted that the Greek general practice data reported by Soler et al. [50] were collected between 2003-2004, while the present study was conducted during 2008. A number of legislative reforms and other interventions aimed at encouraging general practice as a career choice have been introduced during this time [74]. Therefore, further research is required to determine whether the finding of reduced personal accomplishment over time will be replicated in a study synchronously comparing Greek fully licensed general practitioners with general practice residents rotating in secondary institutions or finishing their residency training in primary health centers.

**Table 4:** Descriptive analysis of previously published burnout data in Greek doctors and comparison with the present study.

Studied population	Study authors and publication year	**Burnout rates**^**a**^		Comparable data from the present study		**Comparison**^**b**^	Comments
Internal medicine residents (n = 141) and internal medicine specialists (n = 103) of two Greek public hospitals	Panagopoulou et al, 2006	Internal medicine residents: High EE in 70 (49.6%) High DP in 86 (61.0%) Internal medicine specialists: High EE in 32 (31.0%) High DP in 9 (8.7%)		All residents (n = 311): High EE in 176 (56.6%) High DP in 206 (66.2%)	Internal medicine residents (n = 39): High EE in 23 (59.0%) High DP in 30	No significant differences in burnout rates between the present study's residents (both total and internal medicine subgroup) and Panagopoulou et al.'s internal medicine residents were observed (*P*-values > 0.05). Burnout rates in the present study's residents were significantly higher compared to the internal medicine specialists group (*P*-values < 0.001)	Panagopoulou et al. did not include data on PA
Employees (n = 58) of two Pediatric Oncology Units and a Bone Marrow Transplantation Unit in a Greek children's hospital	Liakopoulou et al, 2008	High EE in 24 (41.4%) High DP in 5 (8.6%) Low PA in 11 (19%)		High EE in 176 (56.6%) High DP in 155 (49.8%) Low PA in 147 (47.3%)		The present study showed increased rates of high EE (P = 0.033), high DP (P < 0.001) and low PA (P < 0.001)	Liakopoulou et al. used different criteria to define high DP (scores ≥ 13) and low PA (scores ≤ 31). For comparison purposes, we adjusted our data to these criteria. The participants of this study were a heterogeneous group that included doctors, nurses and psychosocial staff
Family doctors of 12 European countries (n = 1393) including Greece (n = 45)	Soler et al, 2008	All participants High EE in 599 (43%) High DP in 492 (35.3%) Low PA in 445 (32%) Greek general practitioners High EE in 14 (31.1%) High DP in 33 (73.3%) Low PA in 41 (91.1%)		All residents (n = 311): High EE in 176 (56.6%) High DP in 206 (66.2%) Low PA in 182 (58.5%) Greek general practice residents (n = 23) High EE in 11 (47.8%) High DP in 14 (60.9%) Low PA in 13 (56.5%)		Compared to the total number of family doctors surveyed in 12 European countries, Greek residents showed significantly increased rates of high EE (P < 0.001), high DP (P < 0.001) and low PA (P < 0.001) while the Greek general practice residents had similar EE rates (P > 0.05), higher DP rates (P = 0.011) and increased rates of low PA (P = 0.013). Compared to the Greek arm of the study, Greek residents had significantly increased rates of high EE (P = 0.001), similar DP (P > 0.05) reduced rates of low PA scores, while Greek general practice residents had similar rates of EE and DP (P > 0.05) but reduced rates of low PA (P < 0.001)	

^**a **^EE, Emotional Exhaustion; DP, Depersonalization; PA, Personal Accomplishment

^b ^Comparisons were performed using Pearson's chi-square tests

### Limitations and strengths

The present study's limitations include the cross-sectional design which prevented determination of causality. Furthermore, training satisfaction was documented by questions constructed by the authors based on a review of the relevant literature [44-46] and not with a validated instrument. While the response rate was considerably higher than what is typical of physicians [75], the possibility of response biases cannot be excluded. In order to fully protect residents' anonymity, we were unable to collect any further data on non-respondents. Residents with burnout symptoms may have been less willing to complete and return the questionnaire. Thus, the prevalence of overall burnout could range from 39.5% if all 89 non-respondents were not burnt out, to 60.8% if all non-respondents were burnt out. However, even if the first scenario is true, a burnout prevalence of 39.5% is approximately two-fold higher compared to medical residents from other European Union countries [5,8,54].

Our study also has a number of strengths. It is a large, multi-institutional study in a wide range of residency programs, conducted in three geographically distant Greek cities, although it must be noted that our population was biased towards large centres, each a capacity of over 200 beds. Moreover, we applied a validated and widely used instrument to measure resident burnout, which allowed direct comparisons with different populations and with other samples of medical residents and physicians. Our burnout data are consistent with previous reports, and the present study is the first to document Greek residents' training satisfaction, opinions on the EWTD and job insecurity, as well as being the first to correlate these factors with burnout dimensions.

## Conclusions

In conclusion, the present study provided data on the prevalence of burnout syndrome among Greek residents and their dissatisfaction with residency training and job insecurity, both of which were associated with burnout subscales. Furthermore, this study documented the predominant support and anticipation of the EWTD by Greek residents, although opinion on the EWTD was not associated with resident burnout scores. Considerable moderation of Greek resident burnout levels, complemented with substantial improvements of their training satisfaction, are needed in order to enhance Greek health care quality as well as decrease the incidence of medical errors. The present study indicates that the gradual limitation of working hours will be a short-term measure that may not significantly alleviate resident burnout levels if it is not supplemented by concurrent long-term reforms of residency training in combination with novel patterns of care management, stress reduction programs and other systemic interventions.

## Competing interests

The authors declare that they have no competing interests.

## Authors' contributions

PM and NCK conceived and coordinated the study and drafted the paper; PM carried out the mathematical analysis; AT, DK, NS, NP and ET collected data and assisted in the development of the draft of the manuscript; CL supervised the project and contributed to the draft of the manuscript. All authors read and approved the final manuscript.
